# The Centennial Trends Greater Horn of Africa precipitation dataset

**DOI:** 10.1038/sdata.2015.50

**Published:** 2015-09-29

**Authors:** Chris Funk, Sharon E. Nicholson, Martin Landsfeld, Douglas Klotter, Pete Peterson, Laura Harrison

**Affiliations:** 1 US Geological Survey National Center for Earth Resource Observation & Science (EROS), Sioux Falls, South Dakota 57198-0001, USA; 2 UC Santa Barbara Climate Hazards Group, Geography Department, UCSB, Santa Barbara, California 93106-4060, USA; 3 Earth, Ocean and Atmospheric Science, Florida State University, Tallahassee, Florida 32306-4520, USA

**Keywords:** Climate change, Climate-change impacts, Water resources, Hydrology

## Abstract

East Africa is a drought prone, food and water insecure region with a highly variable climate. This complexity makes rainfall estimation challenging, and this challenge is compounded by low rain gauge densities and inhomogeneous monitoring networks. The dearth of observations is particularly problematic over the past decade, since the number of records in globally accessible archives has fallen precipitously. This lack of data coincides with an increasing scientific and humanitarian need to place recent seasonal and multi-annual East African precipitation extremes in a deep historic context. To serve this need, scientists from the UC Santa Barbara Climate Hazards Group and Florida State University have pooled their station archives and expertise to produce a high quality gridded ‘Centennial Trends’ precipitation dataset. Additional observations have been acquired from the national meteorological agencies and augmented with data provided by other universities. Extensive quality control of the data was carried out and seasonal anomalies interpolated using kriging. This paper documents the CenTrends methodology and data.

## Background & Summary

**Figure 1:**
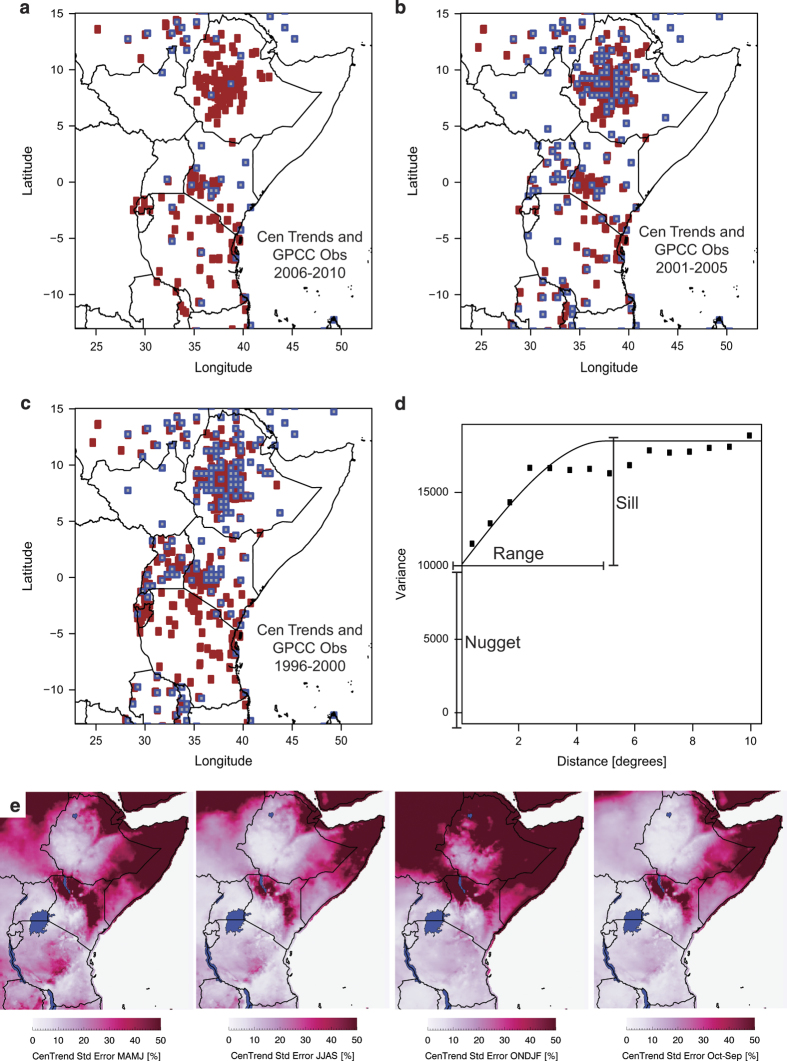
Centennial Trend station locations and standard errors. (**a**–**c**) Centennial Trend (red circles) and GPCC (blue squares) station locations for 2006–2010, 2001–2005, 1996–2000. Symbols are plotted when the location reports for four out of five years. In 2006–2010 the CenTrends (GPCC) data set had 276 (50) observations. In 2001–2005 there were 333 (159) observations. In 1996–2000 there were 404 (187) observations. (**d**) Kriging variogram for March-June e. 1900–2014 average standard errors, expressed as percentages of the 1900–2014 seasonal CenTrends mean fields.

The data set presented here has been developed to support humanitarian relief agencies, East African climate adaptation efforts, and the climate science community’s need for high quality up-to-date rainfall estimates. Over the past 16 years, East Africa has been struck by 8 boreal spring droughts^1^. The 2011 drought resulted in 258,000 deaths^2^ and wide spread food insecurity^3,4^. Rainfall data helped anticipate^5^, monitor^3^ and contextualize^6^ the 2011 dry event. Today, climate scientists in Africa and abroad are working to support better climate prediction and adaptation. Limited station data, however, makes analyzing variability and change at regional scales^7^ difficult. Many products, like the Global Precipitation Climate Centre (GPCC) data set^8,9^ have seen the number of observations fall precipitously since the 1990s^8^. This has produced data gaps over Eastern Africa (Fig. 1a–c). To help overcome these limitations, researchers at Florida State University (FSU)^10–12^ and the US Geological Survey/UC Santa Barbara Climate Hazards Group (CHG)^13–16^ have combined and updated their station archives. The resulting Centennial Trends (CenTrends) data set has substantially better coverage than the GPCC (Fig. 1a–c). The CenTrends moniker does not imply centennial linear trends in African rainfall, but rather a dataset supporting the analysis of seasonal and decadal excursions within a centennial context.

The objective of CenTrends is to provide a reasonably complete and accurate set of gridded seasonal precipitation products for East Africa (−15°S-18°N, 28°E-54° E) from 1900 to 2014 (Data Citation 1). This data can support the activities of the national meteorological agencies, and training workshops like those led by the East African IGAD Climate Prediction and Applications Center (ICPAC, www.icpac.net) and the US Agency for International Development’s Famine Early Warning Systems Network (FEWS NET).

To this end, gaps in the combined FSU/CHG archive have been filled, when possible, with acquisitions from national meteorological agencies. Special attention has been focused on 2000–2014, because global archives provide very few observations over this time periods (Fig. 1a–c), and the character and characteristics of recent decadal East African precipitation variations remains a matter of active interest and debate^1,17–22^.

Extensive quality control of the monthly CenTrends station data has been carried out. This work included screening for extreme values, comparisons with neighboring stations and comparisons with expected values from background climatologies and regional anomalies. The resulting 1900–2014 CenTrends station data set provides the best available regional set of rain gauge data for the Greater Horn of Africa (GHA). Seasonal totals were calculated, translated into anomalies (station value minus long term station means) and interpolated using kriging. Working with station anomalies also limits the errors introduced by inhomogeneous station archives.

Historically, there have been two primary approaches to generalizing in situ observations: regionalization and interpolation. Regionalization breaks Africa into homogenous rainfall areas, and then combines the standardized gauge time series within each region to create a representative index^10,11,23^.

Interpolation is another useful approach that produces gridded maps of data. Climatically aided interpolation^24^ creates grids of precipitation anomalies, based on weighted combinations of neighboring stations. These anomaly grids are then added to background climatologies (long term mean fields) to produce gridded estimates of monthly or seasonal precipitation. Several commonly used global precipitation data sets produced this way are Climatic Research Unit (CRU) archive^25^, the University of Delaware^24,26,27^ dataset and the GPCC^8,9^ product. CHG analyses^14,15,20,22^ have relied on a geostatistical interpolation approach—kriging^28,29^. Here, we show that kriging produces results similar to the GPCC, but with the additional benefit of spatial standard error fields. These standard error fields help us know where uncertainty in the precipitation estimates is high due to low station support. Comparisons of cross-validated CenTrends estimates with estimates based on the GPCC interpolation algorithm and stations indicate substantially better CenTrends performance, with mean absolute errors of ~20% as opposed to 31% for the GPCC.

**Figure 2:**
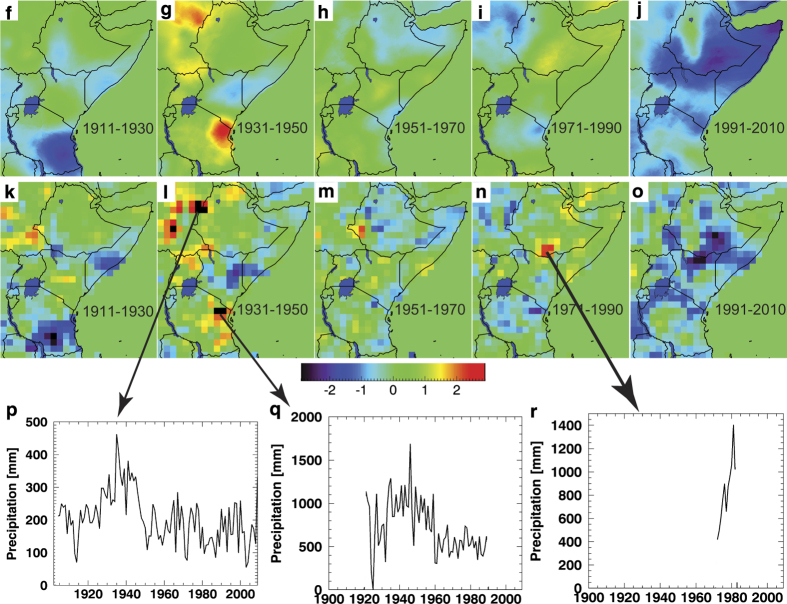
Comparison of standardized anomalies from March-June CenTrends and GPCC data sets. (**a**–**e**) Twenty year average CenTrends anomalies, expressed as standardized deviations. (**f**–**j**) Twenty year average standardized precipitation anomalies, based on GPCC observations interpolated using kriging. (**k**–**o**) Twenty year average GPCC v6 anomalies, expressed as standardized deviations. (**p**–**r**) GPCC data from potentially inhomogeneous station records.

**Figure 3:**
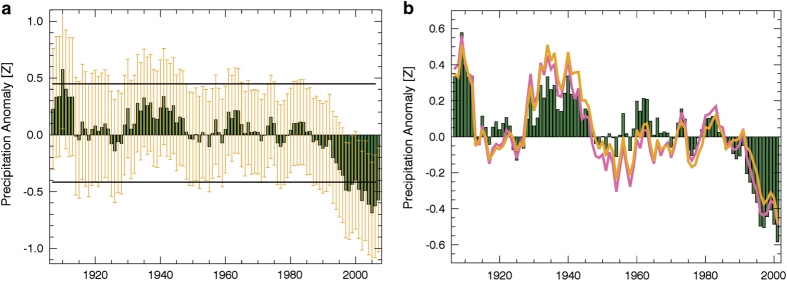
Time series of CenTrends and GPCC precipitation. (**a**) Standardized GHA March-June CenTrends precipitation time series smoothed with 15-year running means. Horizontal bars show boot strapped 90% confidence intervals. Yellow cross-bars show 1 sigma standard errors of the gridded precipitation fields. (**b**) comparison of CenTrends and GPCC precipitation time series (green bars), GPCC v6 gridded data (orange line) and GPCC observations (pink).

We conclude with an example application, plotting CenTrends and GPCC March-June rainfall trends. This example shows how to use standard error estimates and boot strapping to identify low frequency (15-yr) rainfall variations that are statistically significant and well-supported by the observational network. Both the spatial patterns (Fig. 2) and the regional GHA temporal trends (Fig. 3) are quite similar, indicating good performance by the GPCC, and helping to confirm that the recent rainfall declines are not an artifact of reduced station availability.

## Methods

Here we describe the CenTrends rain gauge database, quality control procedures, and interpolation process.

### The CenTrends rain gauge database

The core of the CenTrends data record is a recently updated version of the Nicholson and CHG archives. CHG and FSU scientists combined their datasets, performed a gap analysis, and then acquired additional stations from the Kenyan (76 stations), Tanzanian (60), and Ethiopian (226) Meteorological Agencies. The Kenyan data were used to update the FSU archive. While special focus has been made on acquiring data over the past 30 years, the CenTrends data set covers the 1900–2014 period. Data density over the early part of the 20th and 21st centuries varies substantially by decade and country. The CenTrends interpolation process (kriging) quantifies the uncertainties associated with these changing densities, and in our validation section we describe techniques for quantifying the statistical validity of multi-annual precipitation changes.

The Nicholson East African archive was based primarily on Africa meteorological agency data, augmented with observations from the University of Bergen and Cologne and a historical archive of 217 stations covering the Serengeti region of Kenya and Tanzania. The CHG, archive, on the other hand, is based primarily on global sources: version 2 of the monthly Global Historical Climate Network^30^ (GHCN), the GHCN daily station archive^31^, the National Climatic Data Center’s Global Summary of the Day (GSOD), and the World Meteorological Organization’s Global Telecommunication System (GTS) dataset.

When evaluating GHA precipitation data sets, it is important to remember that the GHA covers 3.7 million km^2^ (Table 1), an expanse equal to half the area of the continental United States. Spread irregularly across this vast region is a sparse network of precipitation gauges, with a spatial distribution determined by history, settlement patterns, and patterns of livelihood (Fig. 1a–c). Station densities tend to follow annual precipitation totals, with moist highland cultivated areas having more gauges, in contrast with the arid almost unmonitored pastoral regions of eastern Ethiopia, Somalia and eastern Kenya. Table 1 lists the average number of monthly CenTrends station observations for the month of May, by country and source, over the 1900–2014 time period. Also shown, for comparison, are the average number of observations from the GHCN monthly archive, the best globally available set of monthly station observations. The CenTrends station data set has more than twice as many observations as GHCN. It should be recognized that the station density of the CenTrends data is still low (1 station per 10,483 km^2^). While we suggest below that the interpolation methods used by the GPCC (inverse distance weighting) produces similar results to the CenTrends’ interpolation method (kriging), the latter approach allows for the calculation of spatially varying error fields. Unfortunately, as noted by the Global Precipitation Climatology Centre^8^, the number of stations being provided by national meteorological agencies and the GHCN has plummeted over the last decade (cf. Fig. 8 in ref. 8). In the GHA, this has manifested as a substantial erosion (Fig. 1a–c) in the number of GPCC ‘observations’ (GPCC v6 0.5° estimates with a least one station observation in that grid cell). There were 187 GPCC observations in 1996–2000, 159 in 2001–2005 and only 50 between 2006 and 2010. In 1996–2000, 2001–2005, and 2006–2010, the CenTrends had 404, 333 and 276 observations in the GHA (Fig. 1a–c). The CenTrends had 2.2, 2.1 and 5.5 times as many observations in these five year periods.

Ethiopia and Tanzania are each roughly the size of Spain and France combined; the Ethiopia 2006–2010 GPCC product had ~4 observations in Ethiopia and ~6 observations in Tanzania. These gaps, and the complete or near absence of robust data records in many areas, underscores the pressing need for better data sharing protocols and agreements.

The same type of comparison between the CenTrends, GHCN and GPCC station densities, extended over time (Table 2), shows the most marked differences during 1981–1999 and 2000–2014, when the GHCN and GPCC densities declined precipitously. The number of GPCC ‘observations’ shown in Table 2 are based on the number of 0.5° GPCC version 6 grid cells that have at least one station observation. While these counts may slightly under-represent the number of GPCC stations, these counts tend to follow the number of GHCN observations closely. The GPCC archive exhibits the same recent decline as the GHCN archive. In Tanzania, Kenya, Ethiopia, Uganda, Rwanda and Burundi the CenTrends archive has substantially more observations. In these periods the CenTrends data set has 3.6 and 28 times as many observations as the GHCN and 2.7 and 4.0 time as many observations as the GPCC v6 archive. Unfortunately, many national GHA observations are not being transmitted to international station archives via systems like the GTS. It should be noted that the CenTrends acquisition process is still a work in progress: gaps remain in Ethiopia prior to 1961, in Uganda, Rwanda, Burundi, Eritrea and Somalia after 1999, in Rwanda, Burundi, Tanzania and Somalia prior to 1921, and in Eritrea prior to 1941. Efforts will continue to improve the FSU/CHG archive.

The final CenTrends station data base is produced through a tiered merging procedure. In the first step, FSU researchers assimilated stations from the large (1400+ stations) FSU African archive, national meteorological agency data sets, and rain gauge observations provided by colleagues at the Universities of Bergen and Cologne. The University of Bergen is part of a Nile Basin hydrologic consortium that updated station records for some stations in the Sudan, Ethiopia, Kenya, Uganda and Tanzania in exchange for the FSU archive for these countries. FSU also obtained a data archive for the Serengeti that contains 217 gauges in Tanzania and Kenya, with observations covering the period 1902–2001. FSU combined and quality controlled these stations. The CHG researchers then added observations from the GHCN monthly and daily data sets (Table 3). For the final period (2000–2014) the CHG also added a small number of screened and quality controlled stations from the GSOD and GTS networks (Table 3). While these data had little impact on the CenTrends data set, their inclusion helps integrate the CenTrends data set with the CHG’s 1981-present near real time station enhanced satellite-based precipitation estimates^32^. These data will also be used to produce updates to the CenTrends data set (see section 2.8).

Note that there are important differences in the overall coverage between countries. Tanzania and Kenya have relatively dense observation networks stretching back to 1900, especially in the Serengeti region. Ethiopia, on the other hand, has very few available stations available before 1961. After 1961, however, an active meteorological agency and accessible data sharing policy provide good coverage through 2014. Coverage in Eritrea and Somalia is limited in general.

### CenTrends quality control procedures

The CenTrends station data were quality-controlled by researchers at FSU and the CHG. Potential data issues were identified based on: i) comparison with climatological means, ii) the identification of extreme standardized precipitation index values^33–35^, iii) the identification of extremely wet monthly observations, iv) the identification of potentially questionable zero values during rainy months, and v) the identification of stations exhibiting low correlation and covariance with neighboring stations or with the rainfall series for the geographical region in which they lie. These issues were examined visually, through spatial and temporal plots of station data. Potential 1981–2014 anomalies were also examined through visual comparison with the Climate Hazards group Infrared Precipitation (CHIRP) imagery^32^. Comparison with CHIRP helped identify both problematic low observations, especially zero values during months with heavy satellite-observed convection, as well as probably correct extreme wet observations—very high monthly rainfall totals accompanied by high satellite-observed convection or similarly extreme values at neighboring stations.

Daily GHCN, GSOD, and GTS data were converted to monthlies if at least 85% of the days were complete. The GHCN monthly^30^ and GHCN daily data^36^ undergo extensive quality control procedures at the National Climatic Data Center (NCDC). The CHG added extra levels of scrutiny for daily GSOD and GTS data. Some GSOD data were found to exhibit repeating values on adjacent days. For example, a string of daily observations might be: 0.0000, 0.0000, 87.653, 87.653, 87.653, 0.0000, …. If a monthly GSOD record had either three or more duplicates, or duplicates totaling to more than 30 mm, the monthly record was omitted. The probability of five digits being the same two or three times is small.

The daily GTS and GSOD values underwent additional screening to flag potential missing values coded as zero. This is a substantial problem with both of these information sources, and can produce completely erroneous ‘droughts’ in the midst of a rainy season, as missing data are coded as zeros and passed through the automated GSOD and GTS networks. If the daily GSOD and GTS values were zero for a given day, but the daily CHIRP precipitation was above the long-term (1981–2014) average daily rainfall intensity, that daily station value was treated as missing. Additional checks were made for spurious zero values for the monthly accumulations. If the station reported a zero monthly value in the GTS or GSOD, but CHIRP indicated 20 mm or more, that station data was treated as missing. Finally, the GTS and GSOD were only used to fill in the last part of the data record (2000 onward), and were only used when higher quality data were not available. The actual number of GTS/GSOD observations used was very small (Table 3).

### Climatologically assisted interpolation of seasonal anomalies

The CenTrends dataset is based on a Climatologically Aided Interpolation (CAI) approach^24^. CAI breaks a climate field into two components: a static background climatology, and a time varying anomaly surface. In this application (which works with seasonal or annual totals), we can express an individual CenTrends grid as the sum of these two components:

(1)C=R¯+I

Where ***C*** represents an nx×ny matrix of CenTrends precipitation values, $\bar{R}$ is a matrix of long term average rainfall, and ***I*** represents a matrix of interpolated station anomalies. The CAI approach takes advantage of the ‘skill’ inherent in good high resolution climatologies^24^. An additional advantage of the CAI approach is that working with station anomalies, as opposed to raw station totals, mitigates the impacts of changing spatial networks.

In East Africa the effect of changing station network can create large spurious variations due to the sparse number of observations and the complex topography and climate. Orographic effects play a large role^37^, and precipitation can vary radically over short distances. When working with simple precipitation totals, for example, the effect of a wet high elevation station disappearing from an archive can create the incorrect semblance of drought. One approach to mitigate this problem is to use standardized anomalies of long homogeneous station archives grouped into climate regions^10,11,37,38^. Here, we use the CAI technique, because gridded precipitation fields are easier to use for many food security and climate adaptation applications. Sample applications relevant to East Africa have been the identification of food security hot spots where drying, population growth, poverty and agricultural extensification coincide^39,40^ and research linking Kenya warming and drying trends to stunting and malnutrition in Kenya^41^.

Another benefit of the CAI approach is that high resolution climatologies can take advantage of the higher number of long term climate normals, since there are typically many more of these than time-varying monthly observations. The CenTrends analysis uses the Climate Hazards Groups Precipitation climatology (CHPclim) as a background climatological mean state^14,42^. The CHPclim (http://dx.doi.org/10.15780/G2159X) uses moving window regressions to take advantage of local correlations between gauge normals and mean satellite precipitation to produce high resolution rainfall climatologies. In regions like Ethiopia, where sparse data and complex topography combine to create complex season cycles^37^, most precipitation is accompanied by high cumulonimbus clouds^43^. The frequency of these cold cloud tops, which can be observed by geostationary satellites, tracks fairly closely with East African precipitation^43–46^. CHPclim draws on this relationship to produce global climatologies that perform better in the GHA than the most commonly used global climatologies -- the University of East Anglia's Climatological Research Unit (CRU) climatology^47^ and the Worldclim^48^ global climate layers. For example, during Ethiopia’s main growing season comparisons with independent validation data indicated monthly mean absolute errors of about 9 mm month^−1^ for the CHPclim, in contrast with errors of more than 30 mm month^−1^ for the CRU and Worldclim datasets^42^. For this study the 0.05° resolution CHPclim climatology was resampled to 0.1° to keep data sizes reasonable.

### Calculating CenTrends seasonal anomalies

The CenTrends monthly station data was accumulated into four sets of seasonal totals covering the boreal spring ‘long’ rains (March-June), the boreal summer monsoon rains (June-September), the boreal fall ‘short’ rains (October-February), and a set of annual accumulations extending from October-September. Twelve sets of monthly grids were also created. As we discuss below, working explicitly with seasonal totals supports the explicit mapping of seasonal standard errors. Monthly data were also provided, however, to give users flexible access to the data.

Seasonal station totals with at least one missing value were treated as missing. Monthly and seasonal station time series were converted to arithmetic anomalies by subtracting the 1960–1989 mean. The 1960–1989 time period was selected because of its high station density (Table 2).

### CenTrends interpolation procedure—defining the variogram

The CenTrends data set was interpolated using kriging, an optimal geostatistical interpolation technique first developed in South Africa for geological mining applications^28,29,48^. While almost all interpolation techniques are based on linear combinations of neighboring observations, they differ in how they estimate the relevant weighting parameters. The Global Precipitation Climatology Centre, for example, uses a modified version of Shepard’s inverse distance weighting procedure^49^, as implemented in the Spheremap package^24^. The CRU, on the other hand, uses a weighting based on a local tessellation (triangulation) of the rain gauge network^25^. Kriging, on the other hand, bases its interpolation weights on an explicit empirically-derived representation of the relationship between the expected variance between any two locations in space. This relationship is a function of distance. The empirical relationship between distance and variance is first estimated using all possible pairs of observations, binned into distance categories. These observed variances are then fit with a numerical equation that can estimate the variance given the distance between any two points. This function can then be used to define a set of optimal (in a least squares sense) interpolation weights for any given target location and distribution of observations. Perhaps more importantly, this distance decay function can also be used to estimate the standard errors of the kriging estimates.

Figure 1b shows an example variogram, based on the average observed variance between the GHA gauge anomalies for March-June. These values (circles) have been calculated over all the March-June seasons between 1960 and 1989, when the most data was available. The next step in the kriging process fits the empirical variance values using a numerical function of the ‘nugget’, ‘range’ and ‘sill’. This fit is shown with the continuous line in Fig. 1b. The ‘nugget’ refers historically to a piece of ore, the smallest unit of space, and expresses the expected value when the distance goes to a very low value. The high nugget of the CenTrends data probably reflects both issues with the data and the fairly high variability of convective rainfall at small spatial scales. Most of the monthly station data used in the CenTrends data set was obtained as monthly values. The high nugget may be due, in part, to the treatment of missing values within the month in some subset of our data. These missing values may have been ignored, or treated as zeros, resulting in some large discrepancies between neighboring stations.

The range, 5 degrees in this case, reflects the distance at which the expected correlation between any two locations will be zero (~550 km near the equator). The sill specifies the expected variance at this distance, which is identical to the total variance of the original station anomalies. We use these parameters to specify a continuous function specifying the variance as a function of the distance between any two locations: *V*(*d*(***a***, ***b***)). *V*(0) will equal the nugget. *V*(*range*) will equal the sill. Subtracting *V*(*d*(***a***, ***b***)) from the sill gives us the expected covariance between any two locations:

(2)Cov(a,b)=sill−V(d(a,b))

Where *Cov*(***a***, ***b***) is the expected variance between any two locations **a** and **b**. *Cov*(***a***, ***b***) is solely a function of the distance between ***a*** and ***b***, d(***a*, *b***). In linear kriging systems, these covariance estimates are used in manner very similar to multivariate regression, which uses the covariance of the predictors (***X*** ^*T*^ ***X***) and the predictors and predictand (***X*** ^*T*^ ***y***) to find an optimal set of coefficients.

### CenTrends interpolation procedure—ordinary kriging

The covariances defined in equation (2) are used to produce ordinary kriging estimates for each location, based on a set of 20 surrounding stations. Ordinary kriging refers to the simplest form of kriging system. While many other approaches, such as techniques incorporating spatial trend terms are possible, ordinary kriging was selected because it is the most transparent, and explicitly limits the contribution of each station to the range specified by the variogram model. The solution to kriging systems closely resembles those used to determine regression parameters via least squares minimization^50^.

(3)w=C−1s

***w*** represents the solution of the kriging system, the weights of the surrounding neighbors. The final estimated interpolated anomaly (elements of **I** in equation (1)) at a given location will be calculated as $i=w^{T}n\prime$, where n′ denotes a set of neighboring station anomalies. ***C*** is an augmented matrix of n-neighbor+1 by n-neighbor+1 co-variance estimates $C=\left[\begin{array}{cc} Cov(n_{i},n_{j}) & 1\\ 1 & 0 \end{array}\right]$. *Cov*(*n*_*i*_, *n*_*j*_) is determined via equation (2) and the distances between each pair of neighboring points. The operator ***C*** ^−1^ acts to remove the expected covariance among the predictors (stations). ***s*** is an augmented vector of expected covariances between each of the station observations and the location (l) being predicted $w=\left[\begin{array}{c} Cov(n_{i},l)\\ 1 \end{array}\right]$. The closer the station is to the target location, the higher its expected covariance and the higher its weight will be, subject to the influence of ***C*** ^−1^.

While kriging requires an explicit fitting of a variogram model, it is generally held as more reliable than automatic procedures such as the inverse distance weighting used in Spheremap or the triangulation process used in the CRU data set. Kriging, for example, has been used to validate the GPCC dataset^8^ and satellite rainfall estimates in Uganda^46^. The main advantage of kriging is that the variogram, station locations, and target locations can be used to develop explicit estimates of the expected standard errors, based on the density and distributions of the observations.

In summary, the two terms of equation (1) are produced independently. The CHPclim data, based on dense in situ gauge normals, high resolution satellite fields, elevation and moving window regression estimates are used to represent the long term mean climatology. Deviations around this mean are based on the CenTrends gauge observations, interpolated using ordinary kriging. This kriging was carried out in the R statistical program, using the gstat package developed by Edzer Pebesma (www.gstat.org)^51,52^.

### Code availability

The code used to produce the CenTrends data set was written by Chris Funk and is freely available on dryad along with the CenTrends data (Data Citation 1).

### Planned update schedule and naming protocol

We plan on updating the CenTrends data set once each year, probably each spring. Data from globally available sources (GHCN, GSOD, and GTS) will be augmented, if possible, by data acquired from national meteorological agencies. The year will be appended to the version number (1) to represent these sequential updates (CenTrends v1 will be followed by v1.2015, v1.2016, …. Changes in version number will represent either a change in the algorithm or spatial domain. Prior versions of CenTrends will be archived, and kept publically available. We would hope that version 2.0 covers all of Africa.

## Data Records

Version 1 of the CenTrends data set provides four sets of seasonal East African precipitation totals: March-June, June-September, October-February, and October-September. An additional set of monthly data was also produced. Variograms like that shown in Fig. 1b were fit by hand for each season and month. While 1900–2014 data is used for each season, the number of total seasons varies, with the March-June and June-September time series having 115 years, while the October-February and October-September data sets each have 114. In each case, the anomalies are interpolated to an 0.1° grid, and then combined with CHPclim estimates to produce seasonal accumulations in mm. This resolution was selected because of its common use in drought early warning and agro-climatological applications in the GHA. The domain selected extended from 28°E to 54°E, 15°S to 18°N. Each field has 261 columns and 331 rows. The upper-left corner of these data center on 18°N, 28°E.

Each season’s CenTrends data are provided in a single netcdf format file, following standard CF4 (Climate and Forecast) metadata conventions. Another netcdf file contains the 12 months×115 years of monthly gridded CenTrends fields. This data (Data Citation 1) is hosted on the CHG FTP site in ftp://chg-ftpout.geog.ucsb.edu/pub/org/chg/products/CentennialTrends/ and can also be accessed at http://datadryad.org.

Each individual netcdf file contains five netcdf variables (Table 4): seasonal or monthly precipitation totals [mm], seasonal or monthly precipitation kriging standard errors [mm], time in days since 1900, latitude and longitude. The Usage Notes section (Section 5 below) provides instructions on reading the data and sample applications, showing how two different approaches -- bootstrapping and the kriging standard error fields, can be used to guide the identification of exceptional multi-annual rainfall deviations.

## Technical Validation

This validation section presents three sets of validation results: i) a cross-validated 1900–2014 error analysis, quantifying the at-station accuracy by season, ii) examination of the kriging standard error fields, and iii) a comparison with the GPCC gridded data set. The cross-validation section (4.1) also compares the cross-validated kriging estimates with a cross-validated implementation of the Shepard’s interpolator used in the GPCC data set. This interpolator was also used to estimate data at the CenTrends locations based on the GPCC ‘observations’ (0.5° grid cell values containing at least one GPCC station). These comparisons allow us to examine the accuracy the CenTrends data set, the relative influence of the interpolation approaches, and the influence of the GPCC station densities. All three case studies are based on March-June accumulations.

### Cross-validated error analysis

Using the gstat library’s cross-validation procedure, we estimated the at-station errors from our kriging model using Leave One Out Cross Validation (LOOCV). In this process, each station observation for each year for each season is withheld, and the kriging system solved to estimate the value at that location. This estimation process is demanding, in that it compares the kriged field to point (rain gauge) observations, which have a high variance. This process also, however, preferentially samples areas where there happen to be rainfall gauges, potentially inflating skill estimates. This can be especially important in places like East Africa, where gauge distributions tend to much more heavily sample wetter highland regions (Fig. 1a–c). The cross-validation mean absolute errors, expressed as a percentage of the mean at-station rainfall, are shown in Table 5. Mean absolute errors (MAE) range from 16–21%, or about 56–76 mm over the four month period. The overall level of accuracy is reasonable, given the monitoring challenges in this region. These statistics can be contrasted with estimates based on the GPCC ‘observations’ and the Shepard’s interpolator (Table 5). Here we show errors based on estimates of the CenTrends observations, using the GPCC values as predictors. As with the cross-validation procedure, stations closer than 0.1° were not used as predictors. The GPCC MAEs are substantially higher (110 to 123 mm), corresponding to percent errors of 31–35 percent. For the 2000–2010/14 time period, the GPCC+Shepard’s interpolator mean absolute error (107 mm) was about twice as big as the CenTrends’ error (56 mm).

Cross-validated interpolations of the CenTrends station data, based on Shepard’s interpolator, produced results very similar to the kriged CenTrends error statistics, MAE scores of 70 to 77, MAE% 20 to 22 (Table 6 (available online only)). The CenTrends data set is more accurate than the GPCC, with MAE values of about 70% of the GPCC errors, at least where we have CenTrends data, because the CenTrends has more data coverage in those regions. Cross-validated results based on kriging and Shepard’s algorithm performed similarly. The primary benefit of kriging is its provision of spatial error maps.

### Kriging standard error accuracy assessment

Accuracies were also assessed via kriging standard errors, which are included as fields in the CenTrends data set (Table 4). Maps of these standard errors (Fig. 1c) provide detailed spatial information, which is extremely important in East Africa because the observation networks are so irregular. Examined spatially, these maps of the averaged standard errors, expressed as a percentage of the long term seasonal average rainfall, range from very low (<10%) to very high (>50%). For many applications, however, such as agro-meteorology and hydrologic analysis, stations distributions may be reasonable, since they tend to mirror population centers, cultivated areas, and mountainous runoff-producing highlands. Analysis focusing on arid lands face much greater challenges, in general. Users should take into account CenTrends’ spatial error information when evaluating inter-seasonal and multi-annual rainfall anomalies. This type of information is poorly conveyed by cross-validation, since by definition data sparse areas have so few observations. Average statistics, like the ratio of mean absolute errors and long term mean precipitation (Table 5), may fail to adequately convey this information. Kriging standard errors can be a valuable tool for assessing the spatial pattern of our knowledge.

### Comparison with the GPCC data set

We next compare CenTrends and GPCC March-June accumulations, since this season has exhibited large recent declines^13,14,17,19,20,22,53^. Figure 2 compares twenty year average standardized March-June anomalies of CenTrends (Fig. 2a–e), GPCC ‘observations’ interpolated via kriging (Fig. 2f–j) and GPCC version 6 anomalies, which have been interpolated with Shephard’s algorithm (Fig. 2k–o). The GPCC ‘observations’ used to produce Fig. 2f–j were based on the GPCC version 6 0.5° grid cells having at least one station observation.

In these images, anomalies were first calculated by subtracting the 1900–2010 mean from each season, and then these anomalies were averaged over sequential 20 year periods. These 20-year averages were then standardized by dividing each location by that locations interannual standard deviation, divided by 20^0.5^. This is the expected standard deviation of the twenty year averages, based on the central limit theorem, assuming normal distributions and no year-to-year to correlation. Neither of these assumptions will be strictly true at each location, and the standardization process is mainly meant to assist in our heuristic comparison of the CenTrends and GPCC fields, helping us visually assess where statistically large decadal variations have taken place.

For the central long rains regions: Ethiopia, Kenya, Somalia and Tanzania, the low frequency CenTrends and GPCC changes appear quite similar. Before 1991, there are few large scale changes in precipitation. Following 1991, rainfall declines precipitously, as suggested in earlier studies^13,53^. There are a few isolated regions within the GPCC v6 data that exhibit large pre-1991 variations. Time series of the GPCC station data in these locations (Fig. 2k–m) suggest that these may be related to station data inhomogeneities. For example, time series in eastern Sudan and northeast Tanzania (Fig. 2k,l) exhibit large declines that could be associated with changing station locations in regions of complex topography. One arid location in south central Ethiopia (Fig. 2m) has a brief, but implausibly wet record, given that this is a semi-arid region dependent on pastoral livelihoods. Despite these few isolated differences, the CenTrends and GPCC data sets present similar stories: parts of East Africa have experienced exceptional recent drying. While the extra quality control of the CenTrends stations has likely identified a few problematic stations, overall the performance of the GPCC seems reliable. Note that while we have extracted GPCC ‘observations’ from the 0.5° grid GPCC grid cells containing at least one observation, we do not explicitly incorporate these GPCC observations into CenTrends.

The effect of the difference in the interpolation algorithm can examined by comparing Fig. 2f–j and Fig. 2k–o. The large nugget within the variogram model (Fig. 1b) results in smoother anomaly fields. For the most recent period, the CenTrends data (Fig. 2e), kriged GPCC data (Fig. 2j), and GPCC data interpolated with the Shepard’s algorithm (Fig. 2o) are quite similar. The GPCC is doing a good job capturing the recent declines. The greater gauge density of the CenTrends data helps corroborate this result, while also proving a more detailed description of the spatial errors (Fig. 1).

## Usage Notes

We conclude with a brief example focusing on precipitation time series of the GHA region for March-June. We also compare this time series with a corresponding GPCC precipitation time series.

### Example: Identifying significant multi-year rainfall fluctuations

The focus here is on reading the March-June NetCDF file and extracting an average time series for a large section of the GHA region (30°E-52°E, 10°S-15°N). We also show how bootstrapping can be used to identify statistically significant 15-year deviations, and how the kriging standard error fields can be used to quantify our level of uncertainty. Sample code (cen_trends_example.pro), written in IDL, and available at ftp://chg-ftpout.geog.ucsb.edu/pub/org/chg/products/CentennialTrends/, can be used to produce the plot shown in Fig. 3a. The first part of the code reads the March-June CenTrends precipitation and standard error fields, creates average 1900–2014 time series, and smooths these data with running 15-year means. The CenTrends precipitation, expressed as a standardized anomaly, is shown with green bars in Fig. 3a. During the standardization process the regional CenTrends precipitation was converted to an anomaly (by subtracting the 1900–2014 mean), and then standardized by dividing by the inter-seasonal standard deviation. Thus Fig. 3a shows some large (more than 0.5 sigma) declines in recent rainfall.

Are these declines statistically significant? We can use a non-parametric approach, bootstrapping, to test each standardized fifteen average shown in Fig. 3a against a sampling distribution based on averaged ‘white noise’. This ‘white noise’ sampling distribution is simulated by creating 10,000 15-year random samples from our unsmoothed standardized 115 year time series. Sorting these, we can identify the 5th and 95th percentile values (−0.4 and +0.43). Any fifteen year average within (beyond) these boundaries is likely (unlikely) to happen by chance (at *P*=0.1). These 90% confidence intervals are shown with horizontal lines in Fig. 3a. The minimum observed 15-year value shown in Fig. 3a (−0.62) is significant at *P*=0.99. The recent declines have been statistically exceptional.

But how do we know if our data can support the identification of these potentially large changes? We can visualize the accuracy of our data by estimating, and plotting (yellow bars Fig. 3a) 15-year averaged kriging standard errors. Since the GHA precipitation time series exhibits no serial correlation (r=−0.06) and is very close to normally distributed (*P*=0.06; Shapiro-Wilks normality test) we can take advantage of the central limit theorem to approximate the expected 15-year standardized standard errors over the GHA region as **ε**(15^0.5^σ)^**−1**^ where **ε** is the time series of areally averaged kriging standard errors and σ is the inter-annual standard deviation of the regionally averaged CenTrends rainfall (41 mm). This is the data shown with yellow bars in Fig. 3a.

It should be noted that the yellow bars in Fig. 3a show an estimate of the standard error *at each grid cell*. Assuming the actual errors are spatially uncorrelated, the error of the regional average would be substantially lower^28^. Thus the error bars shown in Fig. 3b are conservative, indicating a statistically large and detectable decline. It should be emphasized, however, that the spatial pattern of this decline is complex (Fig. 2e,j), and that we have virtually no information over many parts of the GHA (Fig. 1c).

### Time series of GHA CenTrends and GPCC March-June rainfall

We conclude with a short comparison of the CenTrends and GPCC time series over the GHA region used in the example above. GPCC GHA and Eastern East Africa time series have been evaluated in several recent studies^1,17,19,20,22^ examining the decline in the East African spring rains. It is important, therefore, to see how well the GPCC data aligns with the CenTrends data set. Figure 3b presents smoothed standardized rainfall time series for the GHA from three sources: i) CenTrends, ii) GPCC v6, and iii) the GPCC station ‘observations’. The GPCC ‘observations’ were derived by sampling the GPCC v6 data set only at 0.5°grid cells that had at least one observation. The GPCC v6 and GPCC v6 observations were converted to anomalies by subtracting the 1901–2010 mean, averaged over the GHA, and divided by the corresponding inter-annual standard deviation. The 15-year smoothed GPCC and GPCC station time series correlated well with the CenTrends results (r=0.88, 0.86). The temporal evolution of these time series is similar to that presented previously, GHA rainfall has declined substantially, with declines beginning in the mid-1980s. Figures 2 and 3b both indicate substantial agreement between the CenTrends and GPCC data sets. Note, however, that while it has been suggested that GHA pluvials and droughts are caused by Pacific Decadal Variability^20,22^, excursions before 1990 are statistically indistinguishable from white noise (horizontal lines, Fig. 3a) and poorly resolved, given the uncertainty of the interpolated data (yellow lines, Fig. 3a).

It should be noted that the exceptional drying trend coincided with unprecedented warming in tropical ocean regions in recent decades. One suggestion is that the exceptional drying is related to the gradient between equatorial western Pacific and central Pacific SST^1,19,54,55^ and warm west Pacific SST^17,56^. West Pacific SST track very closely with predictions from climate change models, and a warming west Pacific and drying East Africa are associated with the first ENSO-residual empirical orthogonal function of global SST and precipitation^56^. Other research has suggested that the drying is not attributable to climate change and that the GHA pluvials and droughts are driven by Pacific Decadal Variability^20,22^. Attributing historical variability in the GHA is a challenge, as rainfall excursions before 1990 are statistically indistinguishable from white noise (horizontal lines, Fig. 3a) and poorly resolved, given the uncertainty of the interpolated data (yellow lines, Fig. 3a). The substantial decline in rainfall in recent decades is particularly compelling compared to this historical context. The drivers, impacts, and future scenarios related to this trend will likely continue to be a topic of extensive investigation.

## Conclusion

This report has described the station data and statistical interpolation process that were used to produce the CenTrends data set. In general, we found the low frequency CenTrends spatial patterns and time series (Figs 2 and 3) to be quite similar to the GPCC. To the best of our knowledge, the recent reported rainfall declines in East Africa are not an artifact of the GPCC's decaying observation network (Fig. 1a), or potential data inhomogeneities (Fig. 2p–r), but rather a robust and large signal that appears quite consistently in both the GPCC and CenTrends data set.

For the most recent decades, however, our examination of the number of globally available stations revealed a dramatic reduction in the number of available observations (Table 2). This problem, unfortunately, does not appear limited to East Africa and also influences the GPCC^8^. Plots of station counts for countries in Africa (http://chg.geog.ucsb.edu/data/chirps/stations/index.html#_africa) and South America, for example, reveal similar declines in many nations. One advantage of the kriging procedure used in the CenTrends data set is that it can allow us to quantitatively estimate and visualize our spatial uncertainty. Unfortunately, maps of these standard errors, and plots of GPCC station locations (Fig. 1) identify huge gaps. The global precipitation observing system is decaying rapidly. Better data sharing could provide better climate services, and greater societal benefits.

## Additional Information

Table 6 is only available in the online version of this paper.

**How to cite this article:** Funk, C. *et al.* The Centennial Trends Greater Horn of Africa precipitation dataset. *Sci. Data* 2:150050 doi: 10.1038/sdata.2015.50 (2015).

## Supplementary Material



## Figures and Tables

**Table 1:** Area of the countries covered by CenTrends and the average number of CenTrends stations used for each country for each month or season.

	**Area (km** ^**2**^)	**CenTrends**	**GHCN**
Tanzania	945,205	109	49
Kenya	582,650	78	32
Ethiopia	1,127,000	75	22
Uganda	236,040	36	24
Rwanda	26,338	27	1
Burundi	27,830	10	1
Eritrea	117,600	4	4
Somalia	637,657	14	10
Total	3,700,320	353	143

**Table 2:** Comparison of the number of stations in the CenTrends and GHCN monthly and daily data sets

	**1900–1920**	**1921–1940**	**1941–1960**	**1961–1980**	**1981–1999**	**2000–2014**
Tanzania CenTrend	13	76	125	209	162	64
GHCN	7	47	63	111	53	4
GPCC	7	47	62	94	55	11
Kenya CenTrend	26	43	62	146	127	62
GHCN	11	19	24	85	43	3
GPCC	15	24	38	73	56	16
Ethiopia CenTrend	4	6	9	112	176	164
GHCN	4	6	9	63	41	4
GPCC	5	9	13	61	72	35
Uganda CenTrend	13	22	55	67	42	8
GHCN	8	13	49	50	17	0
GPCC	8	16	34	35	19	13
Rwanda CenTrend	1	16	29	56	51	5
GHCN	0	1	2	1	1	0
GPCC	1	4	6	7	5	0
Burundi CenTrend	0	7	10	18	23	3
GHCN	0	1	2	1	2	0
GPCC	0	2	3	3	3	0
Eritrea CenTrend	2	4	8	6	2	0
GHCN	2	4	8	6	2	0
GPCC	2	5	8	5	5	1
Somalia CenTrend	4	25	21	20	10	0
GCHN	2	19	16	14	6	0
GPCC	2	18	17	14	19	0
Total CenTrend	63	199	319	634	593	306
GHCN	34	110	173	331	165	11
GPCC	40	125	180	291	223	77
The first value in each cell indicates the average number of CenTrends observations. The second value shows the number of GHCN monthly observations. The third row shows counts for the GPCC v6 data set. The GPCC results for the first and last columns use 1901–1920 and 2000–2010 as start and end years.						

**Table 3:** The average number of station observations available for the month of May over sequential 20 year time periods

	**1900–1920**	**1921–1940**	**1941–1960**	**1961–1980**	**1981–1999**	**2000–2014**
Tanzania CenTrend	13	76	125	209	162	64
GHCN	4	24	33	67	28	3
GSOD+GTS						2
Kenya CenTrend	26	43	62	146	127	62
GHCN	11	17	21	36	42	14
GSOD+GTS						6
Ethiopia CenTrend	4	6	9	112	176	164
GHCN	4	6	9	52	32	2
GSOD+GTS						2
Uganda CenTrend	13	22	55	67	42	8
GHCN	6	9	6	6	6	0
GSOD+GTS						0
Rwanda CenTrend	1	16	29	56	51	5
GHCN	0	1	2	0	1	0
GSOD+GTS						0
Burundi CenTrend	0	7	10	18	23	3
GHCN	0	1	2	1	2	0
GSOD+GTS						0
Eritrea CenTrend	2	4	8	6	2	0
GHCN	2	4	8	6	2	0
GSOD+GTS						0
Somalia CenTrend	4	25	21	20	10	0
GCHN	2	5	5	5	3	0
GSOD+GTS						0
The first value in each cell indicates the average number of monthly CenTrends observations. The second value shows the number of GHCN monthly observations used in CenTrends. The third row for each country shows the number of GTS and GSOD stations used.						

**Table 4:** NetCDF data file specification for the CenTrends v1.0 dataset

**Variable**	**Name**	**Longitudes**	**Latitudes**	**Ntime**	**Type**	**Units**
precipitation	pr	261	331	114 or 115	float	[mm]
standard errors	se	261	331	114 or 115	float	[mm]
latitude	lat		331		float	[deg]
longitude	lon	261			float	[deg]
time	time			114 or 115	long	[days since 1900]
Five files are available: CenTrends_v1_MarJun.nc, CenTrends_v1_JunSep.nc, CenTrends_v1_OctFeb.nc, CenTrends_v1_OctSep.nc, CenTrends_v1_Monthly.nc.						

**Table 5:** March-June validation results for the CenTrends kriging process and Shepard's interpolator

**[%]**	**1900–1920**	**1921–1940**	**1941–1960**	**1961–1980**	**1981–1999**	**2000–2014**
*Kriging+CenTrends Data*						
MAE/Mean [%]	21	20	20	20	21	16
MAE [mm]	76	72	70	68	73	56
						
*Shepard’s Interpolator+GPCC Data*						
MAE/Mean [%]	35	35	31	32	31	31
MAE [mm]	123	123	110	115	110	107
						
*Shepard’s Interpolator+CenTrends Data*						
MAE/Mean [%]	23	21	21	20	21	22
MAE [mm]	81	75	73	70	76	77
						
100*MAE/MAE [%]						
Kriging/GPCC [%]	61	58	63	59	67	70
Kriging/Shep [%]	94	95	95	96	97	97
CenTrends kriging errors are based one take-one-out cross-validation. The Shepard's interpolation results are based on GPCC 0.5° observations interpolated to the station archive locations. The third set of statistics compares CenTrends observations with cross-validated estimates based on CenTrends data and the Shepard interpolator. The 2nd to last row reports the relative performance of the Kriging+CenTrends Data and Shepard's Interpolator+GPCC Data experiments. The final row compares the Kriging+CenTrends Data and Shepard's Interpolator+CenTrends Data experiments. The GPCC Data results are accumulated over 1901–1920 and 2000–2010.						

**Table 6:** Kriging standard error values for the GHA region

**Std Error/Mean [%]**	**1900–1920**	**1921–1940**	**1941–1960**	**1961–1980**	**1981–1999**	**2000–2014**
March-June	29	24	23	21	23	28
October-September	42	35	33	29	33	40
October-February	32	25	24	20	22	26
June-September	17	15	14	14	15	16
Each season's errors are expressed as a percentage of that season's mean GHA rainfall.						
