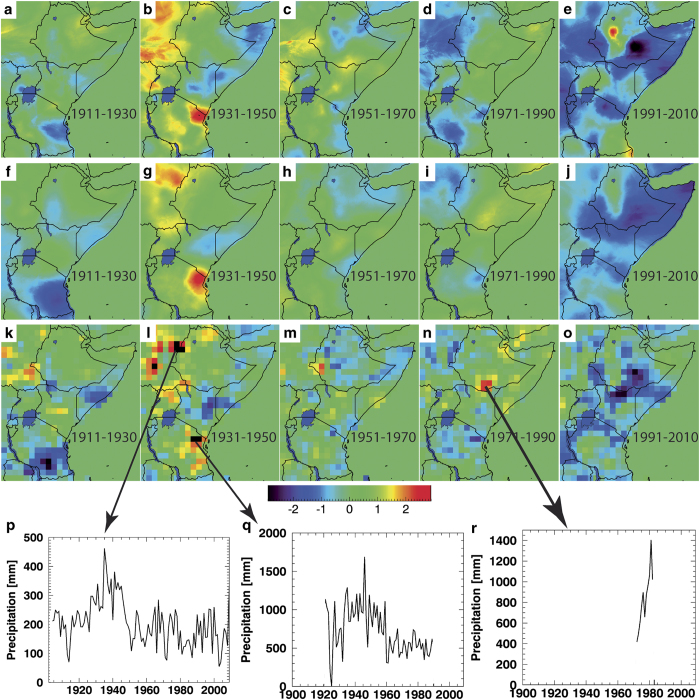# Corrigendum: The Centennial Trends Greater Horn of Africa precipitation dataset

**DOI:** 10.1038/sdata.2018.121

**Published:** 2018-07-03

**Authors:** Chris Funk, Sharon E. Nicholson, Martin Landsfeld, Douglas Klotter, Pete Peterson, Laura Harrison

*Scientific Data* 2:150050 doi: 10.1038/sdata.2015.50 (2015); Published 29 September 2015; Updated 3 July 2018.

Panels a-e were accidentally omitted from Figure 2. A complete version of this figure has been provided below.

## Figures and Tables

**Figure 1:**